# Metabolic Profiling of Mice with Deletion of the Orphan G Protein-Coupled Receptor, GPR37L1

**DOI:** 10.3390/cells11111814

**Published:** 2022-06-01

**Authors:** Margaret A. Mouat, Brendan P. Wilkins, Eileen Ding, Hemna Govindaraju, James L. J. Coleman, Robert M. Graham, Nigel Turner, Nicola J. Smith

**Affiliations:** 1 Orphan Receptor Laboratory, School of Medical Sciences, Faculty of Medicine & Health, UNSW Sydney, Kensington, NSW 2052, Australia; m.mouat@unsw.edu.au (M.A.M.); b.wilkins@unsw.edu.au (B.P.W.); 2 Molecular Cardiology and Biophysics Division, Victor Chang Cardiac Research Institute, Darlinghurst, NSW 2010, Australia; j.l.j.coleman@gmail.com (J.L.J.C.); b.graham@victorchang.edu.au (R.M.G.); 3 Mitochondrial Bioenergetics Laboratory, School of Medical Sciences, Faculty of Medicine & Health, UNSW Sydney, Kensington, NSW 2052, Australia; eileen.ding@sydney.edu.au (E.D.); h.govindaraju@unsw.edu.au (H.G.); 4 Cellular Bioenergetics Laboratory, Victor Chang Cardiac Research Institute, Darlinghurst, NSW 2010, Australia

**Keywords:** G protein-coupled receptor, orphan GPCR, phenotype, metabolism, GPR37L1

## Abstract

Understanding the neurogenic causes of obesity may reveal novel drug targets to counter the obesity crisis and associated sequelae. Here, we investigate whether the deletion of GPR37L1, an astrocyte-specific orphan G protein-coupled receptor, affects whole-body energy homeostasis in mice. We subjected male *Gpr37l1*^−/−^ mice and littermate wildtype (*Gpr37l1*^+/+^, C57BL/6J background) controls to either 12 weeks of high-fat diet (HFD) or chow feeding, or to 1 year of chow diet, with body composition quantified by EchoMRI, glucose handling by glucose tolerance test and metabolic rate by indirect calorimetry. Following an HFD, *Gpr37l1*^−/−^ mice had similar glucose handling, body weight and fat mass compared with wildtype controls. Interestingly, we observed a significantly elevated respiratory exchange ratio in HFD- and chow-fed *Gpr37l1*^−/−^ mice during daylight hours. After 1 year of chow feeding, we again saw no differences in glucose and insulin tolerance or body weight between genotypes, nor in energy expenditure or respiratory exchange ratio. However, there was significantly lower fat mass accumulation, and higher ambulatory activity in the *Gpr37l1*^−/−^ mice during night hours. Overall, these results indicate that while GPR37L1 may play a minor role in whole-body metabolism, it is not a viable clinical target for the treatment of obesity.

## 1. Introduction

With the worldwide rates and cumulative disease burden of obesity and related comorbidities steadily increasing [1,2,3], it is imperative that potential novel drug targets for the treatment of the underlying causes of metabolic dysfunction are identified. Current preclinical and clinical studies appear to show promising results for centrally acting agents [4,5], among which are ligands for G protein-coupled receptors (GPCRs) such as the melanocortin 4 receptor and cannabinoid receptors. In addition to these well-known receptors, some orphan GPCRs (which lack pairings to endogenous ligands) also have demonstrable involvement in the regulation of feeding, insulin activity and whole-body energy homeostasis [6], and may represent viable and novel therapeutic targets for obesity.

The known physiological effects of GPR37L1 include cerebellar development [7], neuroprotection [8] and involvement in cardiovascular homeostasis [9,10], but as yet a metabolic role for this receptor has not been described. There is some evidence to suggest that GPR37L1 may have an effect on body weight homeostasis: genetic deletion of the receptor most closely related to GPR37L1, GPR37, induced lower body weight in mice [11], which may be relevant given the sequence similarity and pattern of expression in the brain of these two related receptors. More importantly, *Gpr37l1* knockout mice were found to have higher body weight than C57BL/6J control mice at 17–19 weeks of age; however, the control mice were not of the same genetic background and the study was not primarily investigating metabolic differences [12], highlighting a need for these results to be confirmed using adequate controls and a more in-depth investigation.

GPR37L1 is most highly expressed in the brain, with specific enrichment in glial cells [13,14,15,16,17]. Notably, *Gpr37l1* is among the 30 most abundant transcripts detected by RNA-seq of mouse astrocytes at 4 months old, and this abundance is maintained in aged mice [18]. In human and mouse, *GPR37L1*/*Gpr37l1* is present throughout the brain and brainstem with low region specificity [17,19] (Human Protein Atlas GPR37L1 data available from http://www.proteinatlas.org/ENSG00000170075-GPR37L1 (version 21, accessed on 31 May 2022) v21.proteinatlas.org/ENSG00000170075-GPR37L1, accessed on 31 May 2022); Allen Mouse Brain Atlas (Gpr37l1) available from https://mouse.brain-map.org/experiment/show/74724670 (accessed on 31 May 2022) RRID:SCR_002978). RNA-based methods have identified *Gpr37l1* transcript in rodent heart, liver, kidney and gastrointestinal tract [20,21], though protein expression in the periphery could not be confirmed by Western blot or β-galactosidase immunostaining in *Gpr37l1*^Lacz/+^ reporter mice [9]. As GPR37L1 appears to be only lowly expressed in peripheral tissues, we postulated that any effects on body weight are likely to be mediated via central mechanisms of energy homeostasis. Pertinent to this hypothesis, GPR37L1 protein expression has been confirmed in brain regions associated with the regulation of food intake and body weight, including the hypothalamus and medulla oblongata [15]. Furthermore, it is possible that GPR37L1 is a receptor for a neuropeptide hormone, since it shows high phylogenetic similarity to endothelin peptide receptors [14,21] and has predicted chemogenomic similarity to other peptide receptors, including orexin and neuropeptide S receptors [22,23], which are well-established regulators of appetite [24,25,26]. With this in mind, we hypothesised that GPR37L1 may be involved in the central nervous system regulation of feeding behaviour or whole-body energy homeostasis. To investigate a potential metabolic role of GPR37L1, we assigned male *Gpr37l1*^−/−^ mice and littermate wildtype controls (C57BL/6J background) to one of two experimental arms; a high-fat diet (HFD) feeding protocol, or a longitudinal observation study where mice were fed standard chow for 1 year. We found modest differences between the genotypes, suggesting that GPR37L1 plays only a minor role in energy homeostasis.

## 2. Materials and Methods

### 2.1. Ethics Statement

All animal work was performed with appropriate ethics approval for each centre: Victor Chang Cardiac Research Institute (VCCRI) and UNSW Sydney, and in accordance with the *Australia Code for the Care and Use of Animals for Scientific Purposes, 8th Edition* (2013). VCCRI project numbers 16/24 and 19/31 were approved by the Garvan Institute for Medical Research/St Vincent’s Hospital Animal Ethics. UNSW Sydney project numbers 18/131A and 19/166B were approved by the UNSW Sydney Animal Care and Ethics Committee.

### 2.2. Generation and Maintenance of the Gpr37l1 Knockout Mouse Line

Here, we use male *Gpr37l1* knockout mice on a C57BL/6J background (hereafter referred to as “*Gpr37l1*^−/−^”). These have previously been characterised and validated as *Gpr37l1*^−/−^ mice by Western blot and RT-qPCR [9], and have been used previously by our laboratory to investigate the cardiovascular effects of GPR37L1 [9,10]. We have also confirmed the genotype of all experimental mice (Supplementary Figure S1) to verify deletion of *Gpr37l1*. The *Gpr37l1* knockout line was generated using the European Conditional Mouse Mutagenesis Program (EUCOMM) method for conditional gene inactivation using *Cre/loxP* and *Flp1/FRT* gene-trapping [27] described as GOI#1 in Coleman et al. [28]. To generate experimental mice, offspring of heterozygous (*Gpr37l1*^+/−^) matings were genotyped (Supplementary Figure S1) and *Gpr37l1*^+/+^ and *Gpr37l1*^−/−^ mice allocated into cages of 5 mice before they reached 5 weeks of age. Only mice from litters sized between 4–9 mice were used.

Mice were housed in individually ventilated XJ home cages (Allentown Inc., Allentown, NJ, USA) with corncob bedding and environmental enrichment (autoclaved wooden chew sticks and transparent red acrylic hut). Room temperature was maintained at 21 °C ± 1 °C. During the study, the body weight of each mouse was manually recorded at least once per week.

### 2.3. Study Design

**Figure 1:**
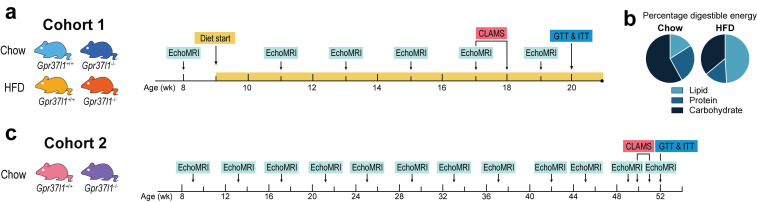
Schematic indicating timeline of experimental interventions. *Gpr37l1*^−/−^ and wildtype littermate control (*Gpr37l1*^+/+^) mice were allocated to 2 cohorts. (**a**) Cohort 1 mice were fed either high-fat diet (HFD) or standard chow for 12 weeks, beginning at 9 weeks of age (*n* = 10 for all groups). Body composition was measured regularly by an NMR–MRI method (EchoMRI). Mice were subjected to respirometry using the Comprehensive Lab Animal Monitoring System (CLAMS) and underwent a glucose tolerance test (GTT) and an insulin tolerance test (ITT) at the ages indicated. (**b**) Macronutrient composition of chow and HFD diets, expressed as the percentage of digestible energy in the diet. (**c**) Cohort 2 was maintained on standard chow until 1 year of age (*n* = 20 for both groups). During this time, body composition was measured using EchoMRI, and GTTs and ITTs were performed at the ages indicated. A subset of cohort 2 mice underwent CLAMS (*n* = 10 *Gpr37l1*^+/+^ and *Gpr37l1*^−/−^).

Figure 1 illustrates the timeline of experimental interventions administered across the two arms of this study. Cohort 1 (chow vs. HFD) was a cohort of male *Gpr37l1*^−/−^ mice and littermate wildtype (C57BL/6J background) controls, which were administered either an HFD or maintained on standard chow for a period of 12 weeks, beginning at 9 weeks of age (*n* = 9–10 per group). These mice also underwent metabolic assessment using indirect open-circuit calorimetry via Comprehensive Lab Animal Monitoring System (CLAMS; Columbus Instruments, Columbus, OH, USA), glucose tolerance tests (GTTs) and insulin tolerance tests (ITTs) close to the end of the diet-fed period. Body weight and food intake of these mice were also measured manually twice weekly.

Cohort 2 consisted of male *Gpr37l1*^−/−^ mice and littermate wildtype controls (*Gpr37l1*^+/+^; *n* = 19–20 per group), which were maintained on standard chow and in home cages until they reached 1 year of age. These mice were subjected to GTT and ITT at 51–52 weeks of age, and a subset of this cohort was also characterised using CLAMS. Body weight and food intake were measured weekly. Body composition was measured for each cohort periodically using the EchoMRI-900.

### 2.4. Mouse Diets and Food Intake Monitoring

Mice were fed either standard chow (16% calories from lipid, 26% calories from protein, and 58% calories from carbohydrate, 2.6 kcal/g, Gordons Specialty Feeds, Australia) or high-fat diet (HFD), formulated in-house, based on rodent diet no. D12451 (Research Diets Inc., New Brunswick, NJ, USA). HFD contained 49% calories from lipid, 15% calories from protein, and 36% calories from carbohydrate, 4.7 kcal/g. HFD was prepared in-house in the following proportions (by weight): 22% lard (code: LARD15, JL Stewart, Glendenning, Australia), 23% casein (Cottee Group, Sydney, Australia), 20.2% sucrose (code: GRAD25B, JL Stewart, Australia), 17% corn starch (code: CFLR25W, JL Stewart, Australia), 5% wheat bran (code: BRAN10UF, JL Stewart, Australia), 3% safflower oil (Proteco Gold, Kingaroy, Australia), 2% gelatin (code: GEL2, JL Stewart, Australia), 4.5% AIN-93M mineral mix (code: 0296040102, MP Biomedicals, Seven Hills, Australia), 1.3% AIN-93-VX vitamin mix (MP Biomedicals, 0296040201, Australia), 1.3% trace mineral mix (MP Biomedicals, 0296026401, Australia), 0.4% choline bitartrate (code: C1629, Sigma, North Ryde, Australia), and 0.3% methionine (code: M9500, Sigma, Australia). Schedules of diet interventions are detailed in Figure 1. Food intake was monitored manually weekly by weighing the remaining food in the food hopper, which was then averaged between the five mice per cage to determine average food consumption per mouse per day. Food intake was calculated based on the caloric density of each diet.

### 2.5. Body Composition Analysis

Body composition (fat and lean mass) measurements were made by an NMR–MRI method using an EchoMRI-900 (EchoMRI, Houston, TX, USA), as described previously [29]. The machine was calibrated using a canola oil standard prior to each session. This method did not require anaesthesia of the animals.

### 2.6. Glucose Tolerance Test

Mice were fasted for approximately six hours prior to the glucose tolerance test (GTT), by placing them into a fresh home cage and giving them only water. Immediately prior to the GTT, tail tip blood was sampled using a glucose test strip (Accu-chek Performa test strips, Roche, Millers Point, Australia) and blood glucometer (Accu-chek Performa II, Roche, Australia), and this sample was considered the time (*t*) = 0 min time point. Mice were then injected intraperitoneally with a 25% *w*/*v* glucose solution (50% *w*/*v* glucose (Phebra, Lane Cove West, Australia), 0.9% NaCl saline solution (B Braun, Melsungen, Germany)) at a dosage of 2 g glucose per kg of lean mass (as measured by EchoMRI). Tail tip blood sampling was repeated at 15, 30, 45, 60, and 90 min post-injection. Water and cage enrichment were provided during the GTT procedure.

Exclusion criteria: tail tipping not possible; increase in blood glucose following glucose bolus of less than 5 mmol/L. For cohort 1, 3 mice were excluded (1 *Gpr37l1*^+/+^ HFD, 1 *Gpr37l1*^+/+^ chow, 1 *Gpr37l1*^−/−^ chow). For cohort 2, a *Gpr37l1*^−/−^ mouse was excluded due to lack of response to glucose and an additional *Gpr37l1*^−/−^ mouse was excluded from the procedure as tail tipping was not possible.

### 2.7. Insulin Tolerance Test

Mice were fasted for approximately six hours prior to the insulin tolerance test (ITT) procedure, by placing them into a fresh home cage and giving water only. Prior to the ITT, tail tip blood was sampled using a glucose test strip (Accu-chek Performa test strips, Roche, Australia) and blood glucometer (Accu-chek performa II, Roche, Australia), and this sample considered the *t* = −10 min time point. Blood glucose was then measured again at *t* = 0 min, immediately prior to injection to account for the glucose spike attributable to initial handling. Mice were then injected intraperitoneally with a 0.125 U/mL insulin solution (human insulin, Actrapid, Novo Nordisk, North Sydney, Australia) in 0.9% NaCl saline solution (B Braun, Germany) at a dosage of 1 U per kg of lean mass (as measured by EchoMRI). Tail tip blood sampling was repeated at 10, 20, 30, 45, and 60 min post-injection. Water and cage enrichment were provided during the GTT procedure.

Exclusion criteria: symptoms of hypoglycaemic shock or continually decreasing blood glucose levels at *t* = 60 min or required rescue by glucose bolus; tail tipping not possible. No mice in cohort 1 were excluded. In cohort 2, one *Gpr37l1*^−/−^ mouse was excluded as tail tipping was not possible.

### 2.8. Indirect Calorimetry

Open-circuit indirect calorimetry was performed on the mice using the Comprehensive Lab Animal Monitoring System (CLAMS) (Columbus Instruments, Columbus, OH, USA) to measure oxygen consumption, carbon dioxide production and movement. Derivative measures (respiratory exchange ratio and heat production) were calculated by integrated software. Centre-feeder cages were used. Locomotor activity was measured by infrared beam breaks within the cages in the XZ plane. Mice were acclimatised to cages for 24 h prior to CLAMS recording, then a 24 h recording period was initiated, with O_2_ and CO_2_ reads every 20 min for cohort 1 and every 10–13 min for cohort 2. Mice were provided with water and powdered diet (chow or HFD) ad libitum for the duration of acclimatisation and recording periods.

### 2.9. Tissue Collection and Morphometry

Mice were euthanised by cervical dislocation. Trunk blood was collected by syringe from the chest cavity. Tissues were collected and weighed immediately after harvest to determine weight of whole heart, liver, inguinal white adipose stores, epididymal white adipose stores, intrascapular brown adipose stores, quadricep, and tibialis anterior. Liver and quadricep were additionally freeze-clamped and stored at −20 °C for later processing and assays.

### 2.10. Triglyceride and Non-Esterified Fatty Acid Assays

Triglyceride content of quadricep and liver tissue and isolated serum were determined using the Roche Triglycerides GPO-PAP kit (ref no. 11730711, Roche, Basel, Switzerland) for cohort 1, and using the Triglycerides (GPO) (Liquid) Reagents Set (ref no. OT932-780 PT, Pointe Scientific Inc., Canton, MI, USA). Assay was performed according to manufacturer’s protocol, using 5 µL of sample (serum or tissue triglycerides) per well.

Triglycerides were extracted from muscle and liver in solvent (2 chloroform:1 methanol) using a Precellys 24 homogeniser (Bertin Instruments, Montigny-le-Bretonneux, France). After 12 h room-temperature incubation with shaking, phase separation was induced by addition of 1 M sulfuric acid. The organic phase was separated, evaporated under nitrogen, and resuspended in 100% ethanol. Due to extraction issues, there was one cohort 1 *Gpr37l1*^−/−^ chow sample excluded from liver triglyceride analysis, and one cohort 1 *Gpr37l1*^−/−^ chow, one cohort 1 *Gpr37l1*^+/+^ chow and one cohort 2 *Gpr37l1*^+/+^ sample were excluded from quadriceps triglyceride analysis.

Non-esterified fatty acid (NEFA) content of serum was determined using the NEFA-C kit (#279-75401) Fujifilm WAKO Pure Chemical Corporation, Japan. Assay was conducted according to the manufacturer’s protocol, using 5 µL of sample (mouse serum).

### 2.11. Statistics and Data Analysis

Column graphs display mean ± SEM, with individual data points represented by open circles. XY plots show mean ± SEM. Statistical tests were performed using in-built analysis in GraphPad Prism version 8.0.2 (GraphPad, San Diego, CA, USA) or IBM SPSS Statistics 26 (IBM, Armonk, NY, USA). For experiments involving 4 groups (HFD vs. chow; *Gpr37l1*^−/−^ vs. *Gpr37l1*^+/+^) with single measurements, ordinary two-way ANOVA was used with the Bonferroni post hoc test, which was substituted for a Kruskal–Wallis test with Dunn’s multiple comparisons where normal distribution was violated. Repeated measures three-way ANOVA with Bonferroni post hoc test was used to analyse data with four groups and multiple repeated data. For experiments with two groups (*Gpr37l1*^−/−^ vs. *Gpr37l1*^+/+^), unpaired two-tailed Student’s *t* test was used; Welch’s correction was additionally used in cases of unequal variance. For non-normally distributed data (as determined by Anderson–Darling and Shapiro–Wilk normality tests), Mann–Whitney test was used. For two-group data with repeating measures, repeated measures two-way ANOVA with Bonferroni’s corrected multiple comparisons was used. For heat production, ANCOVA (with lean mass as covariate) was substituted for ANOVA. In all cases, significance was defined as *p* < 0.05. Minimum *p* values reported by Prism are reported as <0.0001; minimum *p* values reported by SPSS are reported as <0.0005. Power calculations were performed using G*Power 3.1 [30].

## 3. Results

The present study was designed to investigate the metabolic effects of the deletion of *Gpr37l1* in mice, by thoroughly characterising the metabolic profile of *Gpr37l1*^−/−^ mice compared with wildtype (*Gpr37l1*^+/+^; C57BL/6J background) littermate controls. We have previously characterised this *Gpr37l1* knockout mouse line and used it in studies investigating the cardiovascular effects of GPR37L1 [9,10], and this study investigates heretofore unexplored metabolic roles for this receptor. We chose to approach this question from two angles, using a high-fat diet (HFD) feeding protocol to investigate possible differences in the onset of obesity in these mice (Figure 1a,b), alongside a longitudinal cohort to investigate any changes in body composition over one year (Figure 1c).

### 3.1. High-Fat Diet Induces Obesity in Gpr37l1^−/−^ Mice

**Figure 2:**
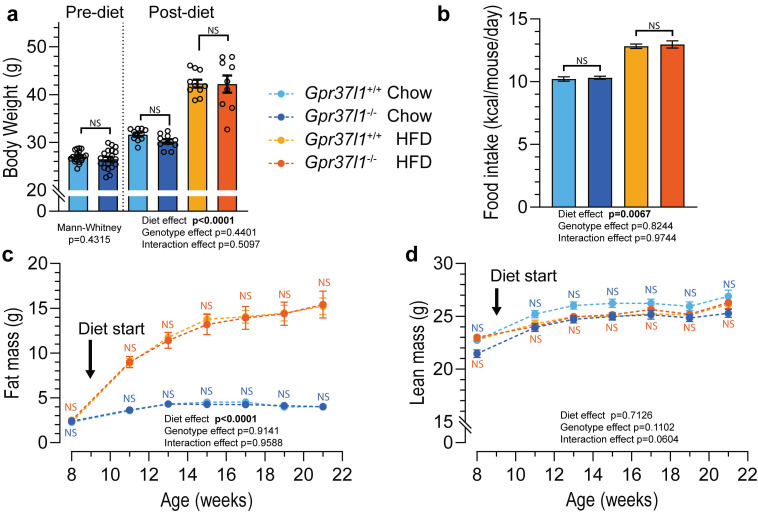
Effect of high-fat diet on *Gpr37l1*^−/−^ and *Gpr37l1*^+/+^ mice. Male mice lacking *Gpr37l1* (*Gpr37l1*^−/−^) and wildtype (*Gpr37l1*^+/+^; C57BL/6J background) littermate mice were allocated to receive standard chow or high-fat diet (HFD) for a period of 12 weeks, from 9 weeks of age. (**a**) Body weight before starting the diet (9 weeks old) and post diet (21 weeks old) was measured manually using an analytical balance. (**b**) Food intake of each cage (5 mice) was manually measured twice weekly. Body composition measurements were performed at regular intervals to determine fat mass (**c**) and lean mass (**d**) of all mice. Solid circles and histograms indicate mean; error bars indicate SEM. Open circles represent individual subject values. Data analysed by Mann–Whitney test (**a** pre-diet), two-way ANOVA ((**a**) post-diet), repeated measures two-way ANOVA (**b**) or three-way ANOVA (**c**,**d**) with Bonferroni post hoc test at each time point. Significance was defined as *p* < 0.05 and is denoted by bolded *p* value; NS denotes non significance. *n* = 9–10 subjects in each group (**a**,**c**,**d**); *n* = 2 cages, 9 replicate measures (**b**); ages indicated.

Body weight was not different between *Gpr37l1*^−/−^ and controls prior to commencement of the HFD/chow feeding protocol (Figure 2a). After the 12-week feeding protocol, HFD-fed mice had significantly higher body weight than the chow-fed cohort, with no differences between the genotypes (Figure 2a). As expected, mice fed HFD consumed more calories per day than did the chow-fed cohort, though there was no effect of *Gpr37l1* deletion on average food intake (Figure 2b).

To attribute body weight changes to particular body compartments, body composition was measured by EchoMRI at regular intervals during the feeding period. Mice that were fed an HFD accumulated significantly more fat mass over the course of the study than did chow-fed mice, whose fat mass remained relatively stable (Figure 2c). Consistent with the body weight comparisons, there was again no difference between the *Gpr37l1*^−/−^ mice and their wildtype counterparts in the amount of fat mass accumulation. The lean mass of all groups increased over the course of the study (Figure 2d); while there were no significant effects of diet or genotype, multiple comparisons revealed significant differences in lean mass between chow-fed *Gpr37l1*^−/−^ and wildtype mice.

Corroborating the body composition analysis, the analysis of manually measured tissue weights revealed that HFD caused a higher weight of inguinal, epididymal and inter-scapular brown fat depots compared with chow-fed controls (Table 1), though again, there was no significant effect of *Gpr37l1* knockout. Alongside this, biochemical analysis of tissue triglyceride content in liver and quadriceps muscle indicated elevated triglycerides in the HFD-fed mice compared with chow-fed controls, and no differences between the genotypes (Table 1). The analysis of serum triglyceride and non-esterified fatty acid (NEFA) levels showed higher NEFA content in the serum of HFD-fed animals, without any genotype effect.

### 3.2. Glucose Tolerance and Insulin Sensitivity in HFD-Fed Mice Is Not Affected by Gpr37l1 Deletion

**Figure 3:**
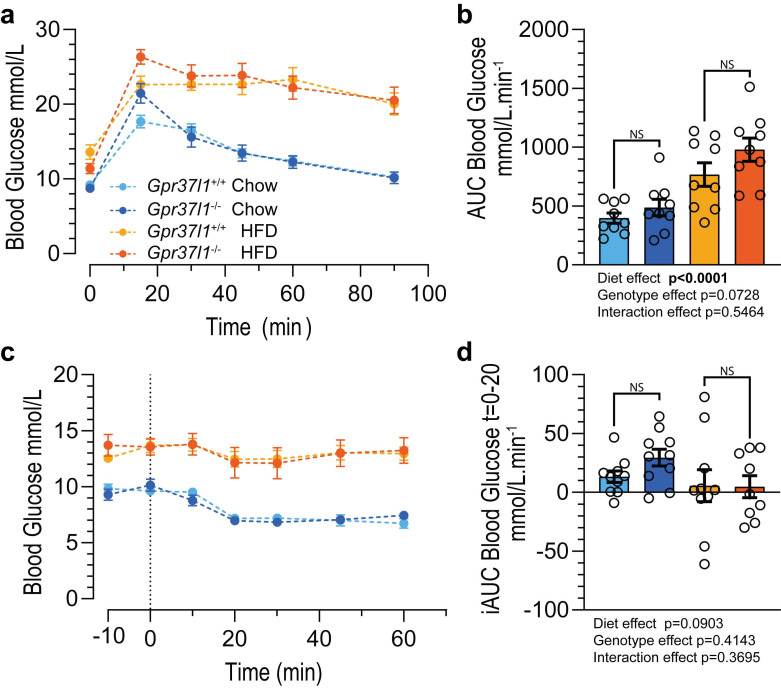
Glucose and insulin tolerance in *Gpr37l1*^−/−^ and *Gpr37l1*^+/+^ mice fed chow or high-fat diet. All mice in the cohort 1 group were subjected to a glucose tolerance test (GTT) and an insulin tolerance test (ITT) following a 12-week period of either chow or high-fat diet (HFD). (**a**) During the GTT, an intraperitoneal glucose bolus (2 g glucose per kg of lean mass) was administered to *Gpr37l1*^−/−^ and wildtype (*Gpr37l1*^+/+^) littermates, and blood glucose was recorded immediately prior to glucose administration (*t* = 0 min) and periodically for 90 min afterwards. (**b**) Response was quantified using area under the curve (AUC) analysis after subtraction of basal blood glucose level for each mouse. (**c**) During the ITT procedure, blood glucose was measured twice prior to intraperitoneal insulin administration (1 U per kg of lean mass) at *t* = 0 min. (**d**) Drop in blood glucose following insulin was quantified using an inverse AUC (iAUC) analysis by subtracting the *t* = 0 glucose value from all timepoints, then calculating AUC between 0 and 20 min. Solid circles and histograms indicate mean; error bars indicate SEM. Open circles represent individual subject values. Data ((**b**,**d**) only) analysed by two-way ANOVA with Bonferroni post hoc test. Significance was defined as *p* < 0.05 and denoted by bolded *p* value; NS denotes non significance. GTT: *n* = 9 all groups. ITT: *n* = 9–10 for all groups, 20–21 weeks old.

To investigate potential genotype differences in glucose handling, as would be expected in metabolic dysfunction, glucose tolerance tests and insulin tolerance tests were performed on cohort 1 mice at 20 weeks of age (11 weeks on diet). As expected, mice fed an HFD had impaired clearance of glucose from the circulation following an IP glucose bolus (Figure 3a), evidenced by a significant diet effect on the area under the curve (AUC) analysis (Figure 3b). Though there may appear to be a trend towards elevated AUC and thus decreased glucose tolerance in *Gpr37l1*^−/−^ mice on both diets (Figure 3b), this difference was not statistically significant.

In accordance with predicted metabolic dysfunction of HFD-fed mice, an insulin tolerance test (Figure 3c) also indicated that HFD-fed mice had impaired insulin tolerance. Blood glucose decrease due to insulin was quantified by inverse AUC analysis during the first 20 min post IP insulin bolus, which indicated no significant effect of diet or genotype (Figure 3d).

### 3.3. Metabolic Profile of Gpr37l1^−/−^ Mice Is Not Different from Wildtype Controls

**Figure 4:**
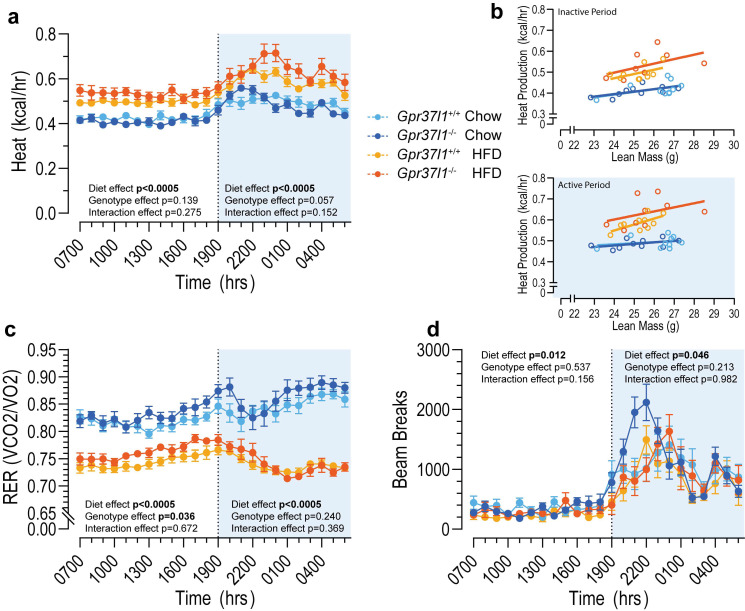
Metabolic profiling of diet-fed *Gpr37l1*^−/−^ and *Gpr37l1*^+/+^ mice by indirect open-circuit calorimetry. At 17–18 weeks of age, and 8–9 weeks of either a chow or high-fat diet (HFD), male *Gpr37l1*^−/−^ and wildtype (*Gpr37l1*^+/+^) littermate mice were subjected to metabolic profiling using the Comprehensive Lab Animal Monitoring System (CLAMS). Mice were acclimated in the system for 24 h, followed by a 24 h period of data collection. Oxygen/CO_2_ readings were taken every 20 min. (**a**,**b**) Heat production and (**c**) respiratory exchange ratio (RER) were derived from these measurements, and were processed into hourly averages. (**b**) Average heat production vs. lean mass for inactive (top panel) and active (bottom panel) periods. (**d**) Physical activity was measured by the number of infrared beam breaks in the XZ plane. Solid circles represent hourly averages for each experimental group; open circles represent individual subjects; error bars indicate SEM. Data analysed by repeated measures three-way ANCOVA (for (**a**); lean mass covariate) or by repeated measures three-way ANOVA (**c**,**d**) within each dark/light period (12 h). Significance was defined as *p* < 0.05 and denoted by bolded *p* value. Time is denoted in standard military time format; light period 0700–1900 (7 a.m.–7 p.m.). *n* = 9–10 subjects per group, 20 weeks old.

To investigate the energy expenditure and substrate utilisation of *Gpr37l1*^−/−^ mice, we subjected all HFD- and chow-fed mice to indirect calorimetry using Oxymax CLAMS (Columbus Instruments, Columbus, OH, USA). This enabled us to derive metabolic heat production (Figure 4a) and respiratory exchange ratio (RER, a measure of substrate utilisation, Figure 4c) from gas exchange rates, as well as activity (Figure 4d) from the number of infrared beam breaks.

To account for the high metabolic demand of lean tissues in the analysis of heat production, lean mass was measured by EchoMRI prior to placing the mice into metabolic cages. Heat production was then analysed by ANCOVA with lean mass as the covariate. Heat production was increased during both the active (dark) and inactive (light) periods (Figure 4a), and RER was decreased over both circadian periods (Figure 4c) in HFD-fed mice versus chow-fed mice. The deletion of *Gpr37l1*^−/−^ appeared to affect the correlation between average heat production and lean mass in the HFD *Gpr37l1*^−/−^ group compared with their wildtype counterparts (Figure 4b), which was reflected in a trend towards a genotype effect during the active period by ANCOVA analysis (Figure 4a). Interestingly, significantly elevated RER within the inactive period was observed in *Gpr37l1*^−/−^ mice compared with wildtype controls (Figure 4c), though this was not evident during the active period.

Ambulatory activity also varied by diet; HFD-fed mice had significantly reduced activity levels compared with chow controls during both active and inactive periods (Figure 4d). There were no significant differences observed between the genotypes during either active or inactive periods, and thus this is unlikely to contribute to the observed genotype effect on inactive-period RER.

### 3.4. Effects of Ageing on Metabolic Profile of Gpr37l1^−/−^ Mice

Given the incidental bodyweight differences between *Gpr37l1*^−/−^ and *Gpr37l1*^+/+^ mice we had previously observed during an unrelated study involving aged mice (unpublished observations), we sought to replicate these in a study more appropriately designed for detecting metabolic differences. For cohort 2, we monitored body weight and food intake regularly from 9 weeks of age to 52 weeks, while housed in home cages with standard chow and water available ad libitum.

**Figure 5:**
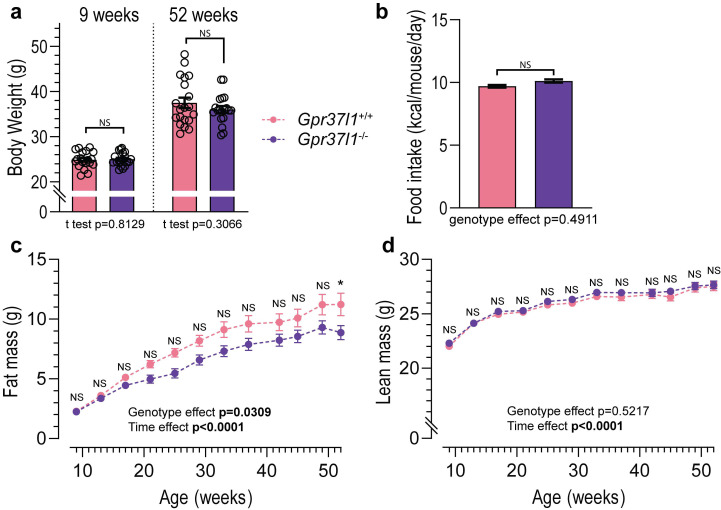
Effect of ageing on body composition of *Gpr37l1*^−/−^ and *Gpr37l1*^+/+^ mice. C57BL/6J male mice lacking *Gpr37l1* (*Gpr37l1*^−/−^) and wildtype (*Gpr37l1*^+/+^) littermates were allowed to age in home cages with standard chow and water available ad libitum. (**a**) Body weight at a young age (9 weeks old) and post ageing period (52 weeks old) were measured manually using an analytical balance. (**b**) Food intake of each cage (5 mice) was manually measured weekly. Body composition measurements were performed at regular intervals to determine fat mass (**c**) and lean mass (**d**) of all mice. Solid circles and histograms indicate mean; error bars indicate SEM. Open circles represent individual subject values. Data analysed by two-tailed *t* test (**a**), repeated measures mixed-effects model (**b**) or repeated measures ANOVA (**c**,**d**) with Bonferroni post hoc test for each time point. Significance was defined as *p* < 0.05 and is denoted by bolded *p* value or by *; NS denotes non significance. *n* = 19–20 subjects in each group (**a**,**c**,**d**); *n* = 4 cages, 40–41 replicate measures (**b**); ages indicated.

The body weight of *Gpr37l1*^−/−^ mice was not different from wildtype controls at 9 weeks of age (Figure 5a). Contrary to our expectations, we did not observe any differences in body weight at 52 weeks of age in *Gpr37l1*^−/−^ mice compared with wildtype mice. There were also no differences in the amount of food intake between the genotypes (Figure 5b). Despite no difference in endpoint body weight, there was significant effect of genotype on fat mass accumulation, and at 52 weeks, *Gpr37l1*^−/−^ mice had significantly lower fat mass than wildtype mice (Figure 5c). Similarly, we observed no differences between *Gpr37l1*^−/−^ and wildtype mice in the accrual of lean mass (Figure 5c) over the study period.

**Figure 6:**
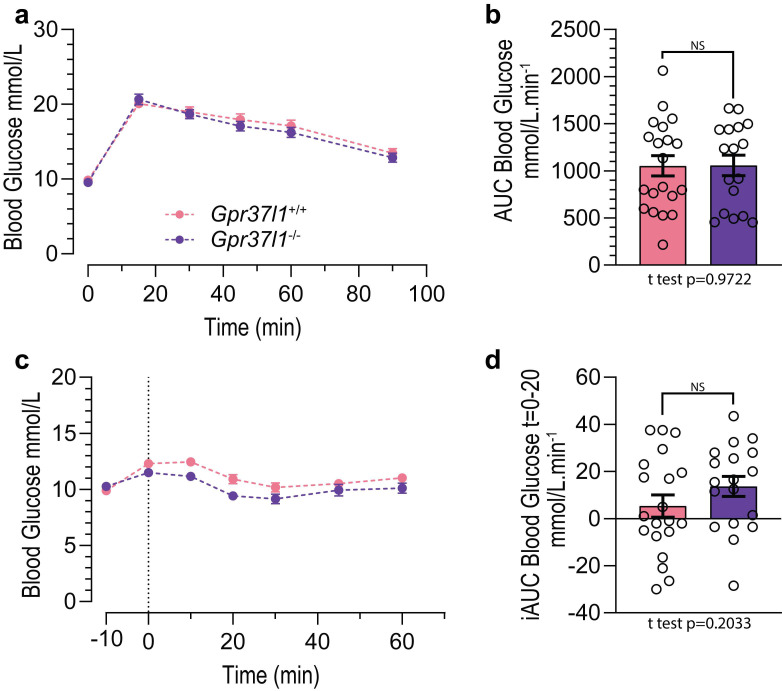
Glucose and insulin tolerance in aged Gpr37l1^−/−^ and *Gpr37l1*^+/+^ mice. All mice in the cohort 2 group were subjected to a glucose tolerance test (GTT) and an insulin tolerance test (ITT) at 51–52 weeks of age. (**a**) During the GTT, an intraperitoneal glucose bolus (2 g glucose per kg of lean mass) was administered to *Gpr37l1*^−/−^ and wildtype (*Gpr37l1*^+/+^) mice, and blood glucose was recorded immediately prior to glucose administration (t = 0 min) and periodically for 90 min afterwards. (**b**) Response was quantified using area under the curve (AUC) analysis after subtraction of basal blood glucose level for each mouse. (**c**) During the ITT procedure, blood glucose was measured twice prior to intraperitoneal insulin administration (1 U per kg of lean mass) at *t* = 0 min. (**d**) Drop in blood glucose following insulin administration was quantified using an inverse AUC (iAUC) analysis by subtracting the *t* = 0 glucose value from all timepoints, then calculating AUC between 0 and 20 min. Solid circles and histograms indicate mean; error bars indicate SEM. Open circles represent individual subject values. Data ((**b**,**d**) only) analysed by two-tailed *t* test. Significance was defined as *p* < 0.05; NS denotes non significance. ITT: *n* = 18 for *Gpr37l1*^−/−^, *n* = 20 for *Gpr37l1*^+/+^; GTT: *n* = 17 for *Gpr37l1*^−/−^, *n* = 20 for *Gpr37l1*^+/+^.

To assess the potential deficits in glucose handling caused by ageing in *Gpr37l1*^−/−^ mice, we also subjected this cohort to GTT (Figure 6a) and ITT (Figure 6c) at 51–52 weeks of age. We observed no difference between the genotypes in their clearance of blood glucose following bolus IP dosing, as indicated by the AUC analysis (Figure 6b). Similarly, there was no statistical difference between the genotypes in the rate of blood glucose drop following IP insulin administration, shown by the inverse AUC analysis of the *t* = 0 to *t* = 20 min period (Figure 6d).

**Figure 7:**
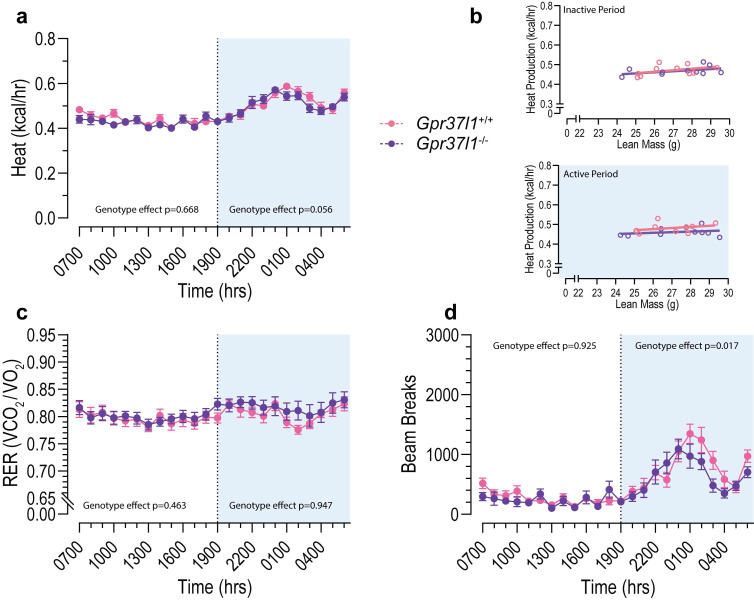
Metabolic profiling of aged *Gpr37l1*^−/−^ and *Gpr37l1*^+/+^ mice by indirect open-circuit calorimetry. At 51 weeks of age, male *Gpr37l1*^−/−^ and wildtype (*Gpr37l1*^+/+^) littermates were subjected to metabolic profiling using the Comprehensive Lab Animal Monitoring System (CLAMS). Mice were acclimated in the system for 24 h, followed by a 24 h period of data collection. Oxygen/CO_2_ readings were taken every 10–13 min. (**a**) Heat production and (**c**) respiratory exchange ratio (RER) were derived from these measurements, and were processed into hourly averages. (**b**) Average heat production vs. lean mass for inactive (top panel) and active (bottom panel) periods. (**d**) Physical activity was measured by the number of infrared beam breaks in the XZ plane. Solid circles represent hourly averages for each experimental group; open circles represent individual subjects; error bars indicate SEM. Data analysed by repeated measures two-way ANCOVA (for **a**; lean mass covariate) or by repeated measures ANOVA (**c**,**d**) within each dark/light period (12 h). Significance was defined as *p* < 0.05 and denoted by bolded *p* value. Time is denoted in standard military time format; light period 0700–1900 (7 a.m.–7 p.m.). *n* = 10 for each group, 20 weeks old.

Finally, a subset of this ageing cohort was subjected to metabolic analysis by indirect calorimetry, and heat production (Figure 7a), RER (Figure 7c) and ambulatory activity (Figure 7d) were derived. The deletion of *Gpr37l1* did not appear to significantly alter heat production during the active or inactive periods, and the relationship between heat production was the same between *Gpr37l1*^−/−^ and wildtype mice (Figure 7b). No genotype effects were observed on the RER of *Gpr37l1*^−/−^ mice versus controls during either circadian period. Though not expected, we did find that mice lacking *Gpr37l1* had lower ambulatory activity during their active period, but this was not observed during the inactive period (Figure 7d). In addition to these measures of energy usage, tissue morphometry and biochemical assays revealed no effect of genotype on the mass of fat depots, muscles, heart or liver, nor on the concentration of tissue or serum triglycerides or serum NEFAs (Table 2).

## 4. Discussion

GPR37L1 is an understudied orphan GPCR [31,32] and its physiological role is not well understood, though it has been previously proposed to have various effects on cardiovascular and nervous systems [7,8,9,10,33,34]. This study represents the first investigation of the potential metabolic effects of GPR37L1. Using male mice with the genetic deletion of *Gpr37l1* (*Gpr37l1*^−/−^) and wildtype littermate controls (*Gpr37l1*^+/+^), we have demonstrated that the knockout of this orphan GPCR induces modest changes to metabolic parameters (respiratory exchange ratio) without alterations to glucose handling in young mice. These differences between *Gpr37l1*^−/−^ mice and their wildtype counterparts were small and did not result in any significant changes in body weight over one year in a longitudinal study. Additionally, we have shown that a high-fat diet (HFD) challenge did not affect *Gpr37l1*^−/−^ mice differently to their wildtype controls.

Though the physiological roles of orphan GPCRs are often not clearly characterised, there are several orphan GPCRs which have been identified to be regulators of energy homeostasis. The gene deletion or silencing of GPR21 [35,36], GPR50 [37], and GPR82 [38] induced lean phenotypes in mice when fed on chow and high-fat diets, and for GPR21 and GPR82 these effects were also associated with improved glucose tolerance. In the opposite direction, the silencing of GPR83 in mice (specifically in the hypothalamic preoptic area) caused a small but significant decrease in central body temperature and higher body weight compared with control mice, despite the same food intake [39]. Like GPR37L1, these orphan GPCRs are highly expressed in the brain, and it was our hope that GPR37L1 would similarly prove to have clinically valuable effects on whole-body energy homeostasis.

We had initially hypothesised that the deletion of *Gpr37l1* in mice would induce metabolic changes given that a *Gpr37l1* knockout line showed differences in body weight compared with C57BL/6J mice [12]. Considering the diffuse expression of *Gpr37l1* in the brain in both mice and humans [13,14], we hypothesised that if *Gpr37l1* were to have a role in metabolic homeostasis, it would likely be via effects on the central nervous system regulation of whole-body energy homeostasis. Though not directly investigated here, we postulated that potential metabolic differences due to *Gpr37l1* deletion may be due to alterations in inflammatory systems; *Gpr37l1* was identified within a predicted, co-regulated protein–protein interaction network (direct or functional interactions) which included proteins associated with inflammation [40], and the closest phylogenetic relative of *Gpr37l1*, *Gpr37*, is known to be involved in inflammation resolution [41,42]. This was an attractive idea, considering that chronic low-level inflammation associated with obesity is increasingly becoming an area of interest for understanding and potentially treating type 2 diabetes [43]. With the ultimate goal of assessing the viability of GPR37L1 as a clinical target for obesity, we sought to assess how the deletion of *Gpr37l1* would affect the whole-body response to HFD challenge, which is known to increase markers of inflammation [44].

Contrary to our expectations, *Gpr37l1*^−/−^ mice gained body weight and fat mass in a similar manner to their wildtype controls when fed HFD. These effects on body weight, fat mass and tissue triglyceride accumulation appear to be in line with other studies using male C57BL/6J mice fed diets with similar fat content [45,46,47,48]. Similarly, both *Gpr37l1*^−/−^ and *Gpr37l1*^+/+^ mice on HFD demonstrated decreased glucose tolerance compared with chow-fed groups, which was expected as C57BL/6J mice are particularly prone to metabolic dysfunction induced by high-lipid diets [49].

Though we did not see large genotype differences in gross body weight in cohort 1, we did observe a significant effect of *Gpr37l1* deletion on RER during the inactive period, indicating a higher relative utilisation of glucose oxidation [50] in *Gpr37l1*^−/−^ mice. Despite effects on RER, the deletion of *Gpr37l1* did not result in differences to energy expenditure that reached the threshold for statistical significance. Nevertheless, it is possible that elevated RER alone can lead to long-term differences in body weight; in the absence of differences in metabolic rate, human studies indicate that elevated RER was a significant predictor of weight gain at follow-up for non-obese subjects [51,52].

Despite these genotype effects on RER and our expectation that these would result in long-term differences in bodyweight, we did not observe significant genotype differences in the longitudinal study of cohort 2 mice. Despite no difference in their endpoint bodyweight, *Gpr37l1*^−/−^ mice accumulated lower fat mass than wildtype mice. The fat mass accumulation rate we observed in the cohort 2 mice is in line with a similar long-term study [46], and glucose intolerance and decreased RER compared with young mice developed as expected [53], though we did not explicitly compare young and aged mice by statistical methods. An interesting observation was that *Gpr37l1*^−/−^ exhibited significantly lower ambulatory activity than wildtype controls during their active period. This was not seen in the younger chow-fed mice in this study, though a previous study in this same mouse line that measured locomotor activity by radiotelemetry reported significantly lower activity in male *Gpr37l1*^−/−^ mice at 14–15 weeks old [10]. These differences in activity between *Gpr37l1*^−/−^ mice and their wildtype controls were not associated with significant genotype effects on energy expenditure or body weight in the aged mice. As an aside, this effect of *Gpr37l1* deletion on locomotor regulation may be related to a motor learning and coordination benefit conferred by *Gpr37l1* deletion [7], although this phenotype is disputed [8]. It is possible, given the expression of GPR37L1 in the central nervous system, that this receptor may be involved in voluntary movement coordination.

Importantly, we only used male mice for this study. This was primarily an ethical decision; this is the first study to investigate a potential role for GPR37L1 in energy metabolism in mice and a potential phenotypic difference in *Gpr37l1* knockout mice was far from certain. Thus, we sought to reduce experimental animal numbers in accordance with the principles of the 3Rs [51]. We elected to use male mice because it is well established that male C57BL/6J mice are much more susceptible than females to dyslipidaemia, weight gain and glucose intolerance in response to high-fat diets [46,54,55,56]. Furthermore, the magnitude of knockout genotype effects in females in metabolic phenotyping studies is often much lower than in their male counterparts [57,58,59,60]. If a scientifically or clinically valuable phenotype had emerged in this study, follow-up experiments with female mice would be justified. Nevertheless, the use of C57BL/6J mice is a strength of this study as they are well characterised in the literature, particularly in their response to high-fat diets [49,61,62], which is in many ways analogous to the disease progression of diet-induced obesity and diabetes in humans [61].

In summary, this study is the first to report a potential role for GPR37L1 in whole-body energy homeostasis. Our results suggest that the deletion of *Gpr37l1* causes very minor alterations to metabolic parameters such as RER without major effects on overall bodyweight in *Gpr37l1*^−/−^ male mice. Furthermore, we observed that food intake was identical between *Gpr37l1*^−/−^ mice and their wildtype counterparts, indicating that it is unlikely this receptor is involved in feeding behaviour. Though the full extent of the physiological functioning of GPR37L1 is yet to be elucidated, our results do not support a role for this receptor in energy metabolism.

## Figures and Tables

**Table 1:** High-fat diet and chow-fed *Gpr37l1*^−/−^ tissue morphometry and triglyceride and NEFA content.

	Chow						HFD						Statistical Analysis		
	*Gpr37l1* ^+/+^			*Gpr37l1* ^−^^/^^−^			*Gpr37l1* ^+/+^			*Gpr37l1* ^−^^/^^−^			Genotype	Diet	Interaction
Tissue Weights (mg)															
Heart	141.39	±	3.44	137.91	±	4.91	136.67	±	2.20	144.16	±	4.93	0.6189	0.8495	0.1781
Liver	1448.63	±	60.78	1305.17	±	56.69	1595.43	±	60.18	1549.70	±	123.93	0.2313	***0.0165***	0.5332
Inguinal WAT (1)	161.93	±	9.08	165.81	±	7.43	769.42	±	60.18	764.73	±	82.54	0.9936	***<0.0001***	0.9313
Epididymal WAT (1)	264.01	±	19.78	261.89	±	17.13	1021.78	±	50.57	1056.57	±	95.62	0.7589	***<0.0001***	0.7289
BAT (2)	87.46	±	3.77	87.71	±	4.37	205.34	±	16.83	201.47	±	14.89	0.8749	***<0.0001***	0.8578
Quadriceps (1)	223.32	±	4.69	223.91	±	4.58	211.10	±	4.57	202.82	±	6.22	0.4472	***0.0021***	0.3818
Tibialis anterior (1)	55.29	±	0.91	54.76	±	1.21	50.65	±	1.30	49.48	±	0.95	0.4508	***<0.0001***	0.7753
**Triglyceride content (nmol/mg)**															
Liver	3.95	±	0.74	4.30	±	0.77	12.90	±	1.99	12.68	±	2.59	0.9716	***<0.0001***	0.8685
Quadriceps	1.98	±	0.33	1.78	±	0.19	3.24	±	0.33	4.76	±	0.74	0.1477	***<0.0001***	0.0615
**Serum Analysis (mM)**															
Triglycerides	1.40	±	0.11	1.17	±	0.15	1.17	±	0.09	1.24	±	0.17	0.5229	0.5625	0.2633
NEFA	0.54	±	0.04	0.53	±	0.04	0.61	±	0.04	0.67	±	0.07	0.5807	***0.0367***	0.5256

WAT: white adipose tissue; BAT: brown adipose tissue; NEFA: non-esterified fatty acids. For fat depots, unilateral (1) or bilateral (2) collection is indicated. Data analysed by two-way ANOVA with Bonferroni post hoc test; significance was defined as *p* < 0.05 and denoted by italicized and bolded font. *Gpr37l1*^+/+^ *n* = 10; *Gpr37l1*^−/−^ *n* = 9–10.

**Table 2:** One-year-old *Gpr37l1*^−/−^ tissue morphometry and triglyceride and NEFA analysis.

	1yo *Gpr37l1*^+/+^			1yo *Gpr37l1*^−/−^			WT:KO
**Tissue Weights (mg)**							
Heart	158.88	±	3.14	153.66	±	2.74	0.2233
Liver	1616.55	±	62.81	1570.95	±	44.99	0.9226 †
Inguinal WAT (1)	470.24	±	55.59	332.61	±	31.49	0.1067 †
Epididymal WAT (1)	663.33	±	59.10	580.68	±	44.87	0.2762
BAT (2)	136.74	±	8.97	136.32	±	9.66	0.8119 †
Quadriceps (1)	210.75	±	2.88	214.08	±	3.76	0.4838
Tibialis anterior (1)	53.97	±	0.68	52.51	±	1.40	0.8621 †
**Triglyceride content (nmol/mg)**							
Liver	12.72	±	2.02	14.67	±	2.34	0.4440 †
Quadriceps	7.20	±	1.09	6.86	±	0.79	0.9880 †
**Serum Analysis**							
Triglycerides (mM)	1.30	±	0.09	1.42	±	0.07	0.2216 †
NEFA (mM)	0.89	±	0.05	0.88	±	0.05	0.8721

WAT: white adipose tissue; BAT: brown adipose tissue; NEFA: non-esterified fatty acids. For fat depots, unilateral (1) or bilateral (2) collection is indicated. Data analysed by two-tailed *t* test, or Mann–Whitney test (marked with †) where normality condition was not met; significance was defined as *p* < 0.05 and is denoted by italicized and bolded font. *Gpr37l1*^+/+^ *n* = 18–20, *Gpr37l1*^−/−^ *n* = 18–20.

## Data Availability

The data presented in this study are openly available in FigShare, DOI: https://doi.org/10.6084/m9.figshare.19656741, accessed on 30 May 2022.

## Supplementary Materials

The following supporting information can be downloaded at: https://www.mdpi.com/article/10.3390/cells11111814/s1, Figure S1: genotyping for Gpr37l1^−/−^ and Gpr37l1^+/+^ mice.
